# Effects of Canola Oil on Hepatic and Cardiometabolic Markers in Non‐Alcoholic Fatty Liver Disease: A Systematic Review and Meta‐Analysis

**DOI:** 10.1002/fsn3.71752

**Published:** 2026-04-19

**Authors:** Kardelen Büşra Ege Gündüz, Sümeyye Bora, Berrak Baştürk, Ceyda Okudu

**Affiliations:** ^1^ Department of Nutrition and Dietetics Haliç University İstanbul Turkiye; ^2^ Department of Molecular Biology and Genetics Atlas University İstanbul Turkiye

**Keywords:** canola oil, liver health, meta‐analysis, NAFLD

## Abstract

Non‐alcoholic fatty liver disease (NAFLD) is closely associated with obesity, insulin resistance, and increased cardiometabolic risk. Dietary fat quality has emerged as a modifiable factor in NAFLD management; however, the specific effects of canola oil on hepatic and metabolic markers remain unclear. This systematic review and meta‐analysis evaluated randomized controlled trials investigating the effects of canola oil consumption in adults with NAFLD. Searches were conducted in PubMed, Web of Science, Scopus, and Cochrane Library databases through December 2024 in accordance with PRISMA guidelines (PROSPERO: CRD42024593566). Random‐effects models were applied to estimate pooled effect sizes. Risk of bias was assessed using the Cochrane Risk of Bias 2 tool, and certainty of evidence was graded using the GRADE framework. Three randomized controlled trials (four intervention arms; *n* = 220 participants) were included. Canola oil consumption was associated with significant reductions in alanine aminotransferase (ALT) (effect size: −0.472, 95% CI: −0.711 to −0.233; *p* < 0.001) and aspartate aminotransferase (AST) (effect size: −0.505, 95% CI: −0.745 to −0.264; *p* < 0.001). Triglyceride levels were also significantly reduced (effect size: −0.593, 95% CI: −1.151 to −0.036; *p* = 0.037), although heterogeneity was substantial. No significant effects were observed for HDL (effect size: 0.154, 95% CI: −0.604 to 0.912; *p* = 0.69) or gamma‐glutamyl transferase (GGT) (effect size: 0.156, 95% CI: −0.173 to 0.486; *p* = 0.352). The certainty of evidence was moderate for ALT and low for AST, triglycerides, HDL, and GGT, primarily because of small sample sizes and clinical heterogeneity. Dietary canola oil may contribute to modest improvements in hepatic enzymes and triglyceride levels in adults with NAFLD. However, the limited number of trials, variability in intervention protocols, and absence of long‐term clinical outcomes warrant cautious interpretation. Larger, well‐designed studies are needed to clarify its role within cardiometabolic risk management strategies for NAFLD.

## Graphical abstract

Summary of the systematic review and meta‐analysis assessing the effects of canola oil on hepatic and cardio metabolic markers in adults with non‐alcoholic fatty liver disease (NAFLD). Based on 3 randomized controlled trials including 220 participants, canola oil was associated with significant reductions in ALT, AST, and triglyceride levels, whereas no significant effects were found for GGT or HDL cholesterol. Overall, canola oil may provide modest benefits for selected NAFLD‐related outcomes, although larger high‐quality randomized controlled trials are required to confirm the evidence.

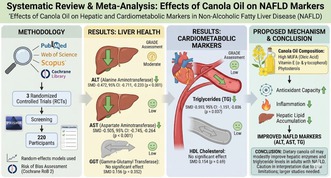

## 1 Introduction

Non‐alcoholic fatty liver disease (NAFLD) is characterized by abnormal hepatic fat accumulation in the absence of alcohol consumption. Due to its rising prevalence and long‐term complications, it has become a major public health concern and is strongly associated with obesity, insulin resistance, and metabolic syndrome. While the global prevalence is approximately 30%, studies from Turkey report higher rates ranging from 48.3% to 60% (Kaya 2019; Younossi et al. 2023). Although histopathology is the diagnostic gold standard, its invasiveness and high cost limit its routine use (Abdelmalek 2021).

Overnutrition is a key contributor to NAFLD development. Excess caloric intake promotes ectopic fat deposition and adipose tissue expansion, accompanied by inflammation and insulin resistance. Increased insulin resistance enhances de novo lipogenesis (DNL), leading to greater synthesis of saturated and monounsaturated fatty acids within very low‐density lipoproteins (VLDLs). This metabolic imbalance induces endoplasmic reticulum stress and oxidative stress in hepatocytes, contributing to inflammation and fibrosis (Heeren and Scheja 2021; Powell et al. 2021). Oxidative stress is also critical in the progression from steatosis to non‐alcoholic steatohepatitis (NASH) (Nagashimada and Ota 2019).

Most individuals remain asymptomatic in the precirrhotic stage, and NAFLD is often detected through elevated liver enzymes such as alanine aminotransferase (ALT), aspartate aminotransferase (AST), and gamma‐glutamyl transferase (GGT). However, these enzymes may remain normal even in advanced disease. NAFLD is frequently accompanied by metabolic abnormalities including dysglycemia, insulin resistance, hyperinsulinemia, dyslipidemia, and hypercholesterolemia (Kupčová et al. 2019).

Since no specific pharmacological treatment is available, lifestyle modification remains the cornerstone of NAFLD management. Healthy dietary patterns and regular physical activity may reduce disease risk and slow progression (Musazadeh et al. 2021). Dietary interventions such as the Mediterranean diet (MD) and ketogenic diet (KD) have shown beneficial effects (Hassani Zadeh et al. 2021; Luukkonen et al. 2020), and higher adherence to the Nordic diet has been associated with lower NAFLD risk (Rasoulizadeh et al. 2024). The New Nordic Diet, based on Scandinavian dietary traditions, uses canola oil as its primary fat source (Mithril et al. 2013). Calorie restriction combined with improved dietary fat quality may reduce hepatic fat accumulation, particularly in populations with low omega‐3 polyunsaturated fatty acid (PUFA) (Musazadeh et al. 2021).

Canola oil contains mainly oleic, linoleic, and palmitic acids, along with α‐ and γ‐tocopherol and other tocopherol derivatives (Matthaus et al. 2016). Due to its high monounsaturated fatty acid (MUFA) content and low saturated fat, it has been proposed as an alternative to extra virgin olive oil (Hoffman and Gerber 2014). Recent studies emphasize the relevance of dietary fat quality in NAFLD, highlighting MUFA‐rich oils such as canola oil (Farhangi et al. 2022; Musazadeh et al. 2021, 2022). USDA data indicate that canola oil provides approximately 60%–65% oleic acid, 17%–25% linoleic acid, and 7%–12% α‐linolenic acid, along with vitamin E components such as 17.3 mg α‐tocopherol and 41.3 mg γ‐tocopherol, which may enhance antioxidant capacity and protect against inflammation, oxidative stress, and hepatic lipid accumulation (Food Data Central Food Details Canola Oil 2019).

Vitamin E has demonstrated beneficial effects in NAFLD, and both AASLD and EASL recommend supplementation (Bril et al. 2019). Meta‐analyses suggest that vitamin E reduces AST and ALT levels and that doses above 600 IU/day may improve fibrosis severity (Ando and Jou 2021; Vogli et al. 2023; Wang et al. 2023). Dietary vitamin E intake has also been associated with a lower risk of NAFLD (Qi et al. 2024).

The increasing use of canola oil has drawn attention not only because of its vitamin E content but also because of its high phytosterol concentration. The total phytosterol content in rapeseed oil (4.6–9.0 mg/g) is approximately twice that in sunflower (2.1–4.5 mg/g) and soybean (2.3–4.7 mg/g) oils (Qi et al. 2024). Given its rich composition of vitamin E, a potent antioxidant, and phytosterols, canola oil consumption is suggested to have therapeutic effects on NAFLD, potentially delaying fibrosis progression. Although canola oil is widely recommended, cautious use has been suggested in certain contexts. For example, one review found moderate increases in waist circumference in specific subgroups despite overall improvements in weight outcomes (Mohtashamian et al. 2025).

Previous meta‐analyses have primarily evaluated the cardiometabolic effects of canola oil in general populations, focusing mainly on lipid profiles and cardiovascular risk markers (Amiri et al. 2020; Pourrajab et al. 2023). However, its role in individuals with established NAFLD, where hepatic steatosis and liver‐specific biomarkers are central outcomes, has not been systematically synthesized. Differences in comparator oils and intervention protocols further limit interpretation. Therefore, a focused evaluation of randomized controlled trials examining canola oil in the context of NAFLD is needed to clarify its hepatic and metabolic implications.

Therefore, this systematic review and meta‐analysis aimed to evaluate the effects of dietary canola oil consumption on hepatic biomarkers and cardiometabolic parameters in adults with non‐alcoholic fatty liver disease (NAFLD), based on evidence from randomized controlled trials.

## 2 Methods

### 2.1 Search Strategy

Our study selection criteria were established on the basis of the PICOS (Participants, Intervention, Comparison, Outcomes, Study type) framework (presented in Table S1), as outlined in the PRISMA (Preferred Reporting Items for Systematic Reviews and Meta‐Analyses) checklist (Page et al. 2021) (presented in Appendix S1). The study protocol was prospectively registered in the PROSPERO international prospective register of systematic reviews (CRD42024593566) prior to study commencement. The keywords “canola oil, rapeseed oil, low erucic acid rapeseed, LEAR, colza oil, *Brassica napus* , NAFLD, nonalcoholic fatty liver, steatohepatitis, steatohepatitides, fatty liver, NASH” were searched in the PUBMED, Web of Science (WOS), Scopus and Cochrane databases until 16 December 2024 by one researcher (K.B.E.G.) (presented in Appendix S2).

### 2.2 Eligibility Criteria

Randomized controlled trials (RCTs), nonrandomized controlled trials, cohort studies or cross‐sectional, observational studies were screened for eligibility. Only studies in which dietary or oil‐based interventions were applied to adults with hepatic steatosis (NAFLD) were included. Although observational and non‐randomized studies were initially screened according to the broader eligibility framework, only randomized controlled trials fulfilled the final inclusion criteria and reported sufficient quantitative outcome data to be synthesized. RCTs in which the mean ± standard deviation (SD) was used to report effect sizes. Meta‐analyses, systematic reviews, and animal and laboratory studies (not involving humans), for example, in vitro studies, were excluded. Two reviewers (K.B.E.G. and S.B.) independently screened the titles and abstracts of all retrieved records using predefined eligibility criteria. Full texts of potentially eligible articles were then evaluated in detail. Discrepancies were resolved by discussion or by consulting a third reviewer.

### 2.3 Quality Assessment of Studies and Data Extraction

Since all studies included in the systematic review were randomized controlled trials, the Cochrane Risk of Bias 2 tool was used for quality assessment, which is composed of the following domains: D1a: randomization process; D1b: timing of identification or recruitment of participants; D2: deviations from the intended interventions; D3: missing outcome data; D4: measurement of the outcome; and D5: selection of the reported result. Two researchers (K.B.E.G. and B.B.) assessed the studies.

Data extraction from the studies selected for inclusion was performed by two researchers (K.B.E.G. and S.B.). The extracted data included the first author's name, the year of publication, the country in which the study was conducted, the study design, sample size, population characteristics, mean age of participants, interventions and controls along with their respective dosages, study duration and type, and the primary outcomes of the study.

### 2.4 Statistical Methods

Comprehensive Meta‐Analysis software (CMA v3; Biostat, Englewood, NJ, USA) was used to perform the quantitative analyses due to its capacity to handle continuous outcomes in studies with small sample sizes. Although RevMan was initially specified in the PROSPERO protocol, the use of CMA did not alter the predefined analytical strategy and was considered a minor methodological adjustment.

Between‐study heterogeneity was assessed using Cochran's *Q* test and quantified with the *I* ^2^ statistic. Given the expected clinical variability across studies including differences in intervention dose, duration, comparator oils, and study populations, random‐effects models were applied for all pooled analyses regardless of *I* ^2^ values. Effect sizes were calculated as weighted mean differences (WMDs) when outcomes were reported in uniform units and as standardized mean differences (SMDs) when measurement scales differed.

Sensitivity analyses were conducted using a leave‐one‐out approach to evaluate the influence of individual studies on pooled estimates. Due to the limited number of included trials (< 10 per outcome), formal statistical tests for publication bias were not considered reliable and were interpreted with caution. A two‐sided *p*‐value < 0.05 was considered statistically significant.

## 3 Results

### 3.1 Study Selection

**FIGURE 1:**
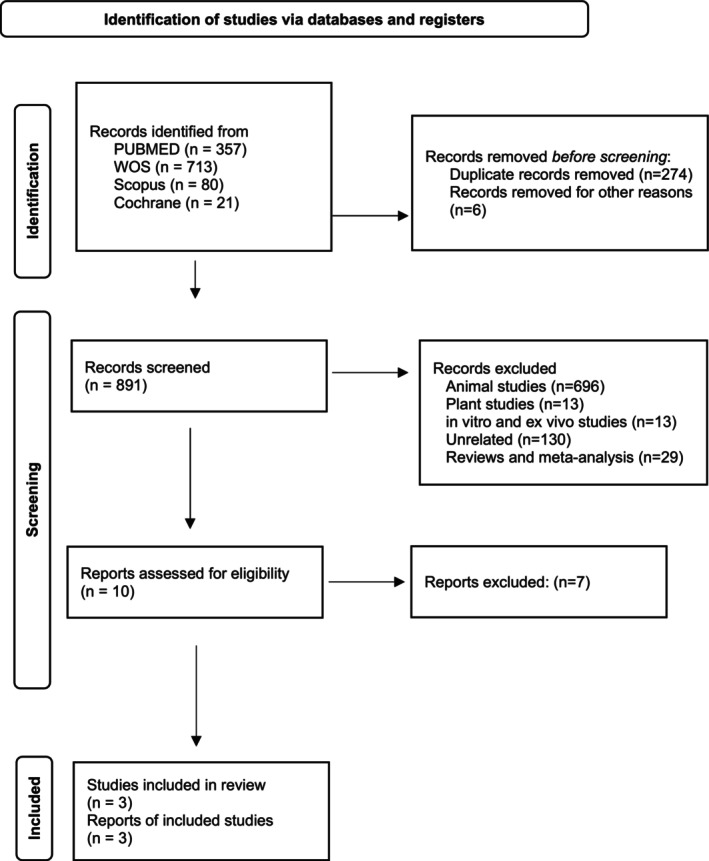
Identification of studies via databases and registers.

357 results were found in the PUBMED database, 713 in WOS, 80 in Scopus and 21 in the Cochrane Library. A total of 1171 studies were found. Duplications were removed with the Rayyan AI‐powered systematic review management platform. In total, 274 of the 1171 studies were duplicates. The results of 6 clinical trial records could not be accessed due to unavailable full texts. A total of 696 studies were animal studies, 29 were reviews, 130 were unrelated to the subject, 13 were plant studies, 12 were in vitro studies, and 1 was an ex vivo study. The remaining 10 studies were identified by two researchers (K.B.E.G. and S.B.), and 5 of them were determined as wrong population, 2 as wrong application. The two reviewers demonstrated high agreement in study selection, with a 90% concordance rate. A PRISMA flow diagram of the study selection process is presented in Figure 1.

### 3.2 Quality Assessment

**FIGURE 2:**
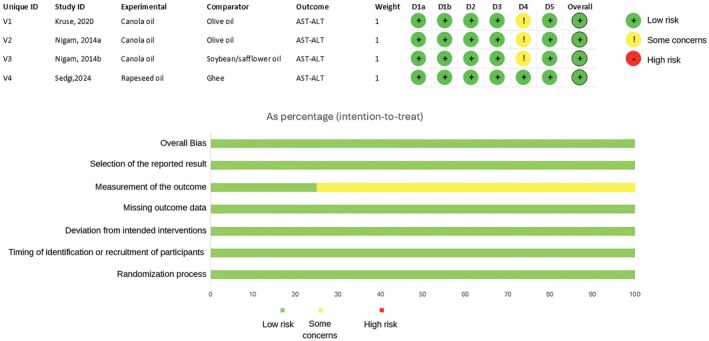
Risk of bias assessment of included studies.

The three studies identified through screening were assessed for quality. In all the studies, the participants were randomly assigned to groups. Additionally, the timing of identification or recruitment of participants was considered low risk. However, in two studies (Kruse et al. 2020) and (Nigam et al. 2014), the reasons for participant dropout were not clearly specified. Only one study (Maleki Sedgi et al. 2024) explicitly stated that the analyst evaluating the data was blinded to the study conditions. Consequently, two studies (Kruse et al. 2020) and (Nigam et al. 2014) raised some concerns in this regard. Overall, two studies were rated as having some concerns, while one study was rated as low risk of bias (Figure 2).

#### 3.2.1 Sensitivity and Influence Analyses

A leave‐one‐out influence analysis was conducted to assess the robustness of pooled estimates for all outcomes. Due to the limited number of included studies, formal statistical tests for publication bias were not performed. The full influence matrix is presented in Table 1.

**TABLE 1:** Leave‐one‐out influence analysis based on Hedges's *g* effect sizes.

Study	Outcome	One study removed					Leave‐one‐out pooled estimate				
		Hedge's *g*	SE	Lower limit	Upper limit	*p*	Hedge's *g*	SE	Lower limit	Upper limit	*p*
Kruse, 2020	AST	−0.584	0.175	−0.927	−0.24	0.001	0.166	0.356	−0.532	0.864	0.641
Nigam, 2014, a	AST	−0.321	0.262	−0.834	0.192	0.220	−0.760	0.252	−1254	−0.266	0.003
Nigam, 2014, b	AST	−0.516	0.252	−1009	−0.023	0.040	−0.210	0.244	−0.688	0.269	0.390
Sedgi, 2024	AST	−0.308	0.257	−0.812	0.197	0.232	−0.744	0.196	−1128	−0.360	< 0.001
Kruse, 2020	ALT	−0.493	0.147	−0.781	−0.205	0.001	−0.297	0.357	−0.988	0.403	0.405
Nigam, 2014, a	ALT	−0.397	0.139	−0.671	−0.124	0.004	−0.716	0.251	−1208	−0.223	0.004
Nigam, 2014, b	ALT	−0.569	0.141	−0.845	−0.293	< 0.001	−0.183	0.244	−0.660	0.295	0.454
Sedgi, 2024	ALT	−0.412	0.176	−0.757	−0.067	0.019	−0.561	0.193	−0.940	−0.183	0.004
Kruse, 2020	GGT	0.028	0.189	−0.343	0.399	0.884	0.633	0.365	−0.081	1348	0.082
Sedgi, 2024	GGT	0.633	0.365	−0.081	1348	0.082	0.028	0.189	−0.343	0.399	0.884
Nigam, 2014, a	TG	−0.736	0.392	−1506	0.033	0.061	−0.289	0.245	−0.768	0.191	0.238
Nigam, 2014, b	TG	−0.714	0.414	−1526	0.098	0.085	−0.332	0.245	−0.812	0.148	0.175
Sedgi, 2024	TG	−0.310	0.173	−0.65	0.029	0.073	−1117	0.204	−1517	−0.718	< 0.001
Nigam, 2014, a	HDL	0.305	0.645	−0.96	1569	0.637	−0.132	0.244	−0.61	0.345	0.587
Nigam, 2014, b	HDL	−0.254	0.15	−0.549	0.04	0.09	0.962	0.257	0.457	1466	< 0.001
Sedgi, 2024	HDL	0.412	0.547	−0.66	1484	0.452	−0.329	0.191	−0.703	0.045	0.084

*Note:* Nigam, 2014,a refers to comparison between the olive oil and canola oil groups; Nigam, 2014,b refers to comparison between the canola oil and safflower/soybean oil groups.

Abbreviation: SE, standard error.

### 3.3 Certainty of Evidence (GRADE Assessment)

The certainty of evidence for each outcome was assessed using the GRADE methodology. The certainty of evidence was rated as moderate for ALT, primarily due to consistent findings and low heterogeneity across studies. For AST, TG, HDL, and GGT, the certainty of evidence was rated as low, driven by small sample sizes, imprecision in effect estimates, and substantial statistical heterogeneity for some outcomes. Overall, the evidence base was limited by small sample sizes, resulting in low‐to‐moderate certainty across outcomes. A summary of the GRADE assessments is provided in Table 2.

**TABLE 2:** Grade evidence profile for canola oil interventions in NAFLD.

Outcome	Number of studies	Study design	Risk of bias	Inconsistency	Indirectness	Imprecision	Publication bias	Overall certainty
ALT	4 RCTs	Randomized trials	Not serious	Not serious (*I* ^2^ = 0%)	Not serious	Serious (small sample)	Not detected	Moderate
AST	4 RCTs	Randomized trials	Not serious	Serious (*I* ^2^ = 60%)	Not serious	Serious	Not detected	Low
GGT	2 RCTs	Randomized trials	Not serious	Serious (*I* ^2^ = 4%)	Not serious	Serious	Undetectable due to small number	Low
HDL	3 RCTs	Randomized trials	Not serious	Very serious (*I* ^2^ = 88%)	Not serious	Serious	Not detected	Low
Triglycerides (TG)	3 RCTs	Randomized trials	Not serious	Serious (*I* ^2^ = 78%)	Not serious	Serious	Undetectable due to small number of studies	Low

*Note:* *I* ^2^: Heterogeneity.

### 3.4 Study Characteristics

Three studies were included in the review. In Nigam's study, participants were divided into two subgroups for more precise comparisons (Nigam et al. 2014). Nigam (2014a) focused on the comparison between the olive oil (OL) and canola oil (CA) groups, whereas Nigam (2014b) compared the CA group and safflower/soybean oil (SA) groups. The characteristics of the included studies are presented in Table 3. All studies had parallel designs. The publication years of the studies ranged from 2014 to 2024. One study (Kruse et al. 2020) implemented an intervention in which canola oil was added for daily consumption, whereas the other two studies (Nigam et al. 2014) and (Maleki Sedgi et al. 2024) replaced dietary fats with canola oil. The intervention durations varied: Kruse conducted an 8‐week intervention, Sedgi implemented a 12‐week intervention, and Nigam carried out a 6‐month intervention.

**TABLE 3:** Characteristics of the participants and interventions of the included studies.

Reference	Study type	Year	Groups (*n*)	Mean age (SD or range)	Health status or clinical condition	Intervention	Intervention duration	Dose	Outcome preintervention (SD)	Outcome postintervention (SD)	Main result
Kruse, 2020, Germany	RCT	N\A	Olive oil (OL): 11 men Canola/rapeseed oil (CA): 16 men	OL: 54 ± 3.95 CA: 58 ± 2.64	Overweight/obese men with NAFLD	Additional 50 g/day oil intervention but participants asked not to change dietary habits	8 weeks	50 g/day	*AST (U/L)* 27.2 ± 2.8 26.88 ± 2.3 *ALT (U/L)* 37.40 ± 5.8 37.69 ± 3.0	*AST (U/L)* 26.04 ± 3.6 26.59 ± 2.8 *ALT (U/L)* 36.28 ± 5.5 35.00 ± 2.2	IHL ↓ in CA group
Nigam, 2014a, India	RCT	May 2007 to December 2009	OL: 30 men CA: 33 men	OL: 37.2 ± 6.2 CA: 38.0 ± 6.4	Men with NAFLD	20 g/day oil intervention as cooking media	6 months	20 g/day	*AST (U/L)* OL: 35.8 ± 21.8 CO: 33.1 ± 13.8 *ALT (U/L)* OL: 39.8 ± 19.9 CO: 33.9 ± 3.2	*AST (U/L)* OL: 33.2 ± 4.6 CO: 29 ± 6.2 *ALT (U/L)* OL: 38.1 ± 11.3 CO: 31.5 ± 6.2	HDL ↑ in OL^*^ TG ↓ CA^**^ Liver fat accumulation ↓ in OL and CA groups Grade I, from 73.3% to 23.3% and from 60.5% to 20%, respectively grade II, from 20% to 10% and from 33.4% to 3.3%, respectively Grade III, from 6.7% to none and from 6.1% to none, respectively
Nigam, 2014b, India	RCT	May 2007 to December 2009	CA: 33 men Safflower/soybean oil (SO): 30 men	CA: 38.0 ± 6.4 SA: 36.2 ± 7.1	Men with NAFLD	20 g/day oil intervention as cooking media	6 months	20 g/day	*AST (U/L)* CO: 33.1 ± 13.8 SO: 31.7 ± 8.9 *ALT (U/L)* CO: 33.9 ± 3.2 SO: 34.7 ± 8.9	*AST (U/L)* CO: 29 ± 6.2 SO: 31.2 ± 13.3 *ALT (U/L)* CO: 31.5 ± 6.2 SO: 34 ± 18.1	
Sedgi, 2024, Iran	RCT	February to August 2022	CA: 55 people Ghee: 55 people	CA: 41.35 ± 9 Ghee: 43 ± 10.1	Patients with NAFLD	Individuals use 3–8 servings (each serving considered as 5 g) ghee asked to replaced rapeseed oil	12 weeks	4–5 servings (5 g per serving)	*AST* 27.5 ± 12.1 30.8 ± 15.8 *ALT* 42.7 ± 31.9 42.1 ± 22.9	*AST* 20.1 ± 6.2 26.7 ± 11 *ALT* 28.3 ± 14.3 37.9 ± 19.13	Steatosis ↓ϯ ALT ↓ GGT↓ Total Cholesterol ↓ TAG ↓, LDL ↓ in CA group

^*^ *p* = 0.004.

^**^ *p* = 0.02.

The dosage of interventions also differed among the studies. Kruse provided an additional 50 g of oil, whereas Nigam used 20 g of oil for cooking. Sedgi, on the other hand, did not specify an exact gram measurement but administered the intervention in 4–5 servings, with one serving size equivalent to 5 g.

### 3.5 Energy Derived From Diet

In the study conducted by Kruse, changes in macronutrient distribution were observed in both the OL and CA groups over the 8‐week intervention period. The total daily caloric intake varied between the groups. In the OL group, the initial daily energy intake of 2255 ± 131 kcal increased to 2417 ± 161 kcal by the end of the study. In contrast, in the CA group, daily energy intake decreased from 2573 ± 210 kcal at baseline to 2322 ± 108 kcal after 8 weeks. In Nigam's study, before the intervention, participants' total energy intake (ln) was 7.6 ± 0.3 kcal in the OL group, 7.5 ± 0.2 kcal in the CA group, and 7.6 ± 0.26 kcal in the SA group. After the intervention, total energy intake was 7.3 ± 0.2 kcal across all groups. In Sedgi's study, only total calorie and fat intakes were reported, while data on the consumption of other macronutrients were not provided. Instead, the study presented information on the intake of food groups such as meat, dairy, fruits, and vegetables. Since the data were shared as figures, exact numerical values were unavailable. However, the study revealed that there was no statistically significant difference in dietary intake between the ghee and CA groups.

### 3.6 Assessment of Liver Fat

The degree of liver fat accumulation in participants was assessed via different methods. Kruse measured the intrahepatic lipid content (IHL), which was evaluated by determining the exact amount of fat via proton magnetic resonance spectroscopy (^1^H‐MRS). In contrast, the other two studies (Sedgi et al. 2024; Nigam et al. 2014) assessed fatty liver grades via ultrasonography, which was performed by an expert radiologist.

In Kruse's study, participants were assigned to groups with initial IHL levels of 13.3% ± 2.5% in the canola oil group and 13.1% ± 1.6% in the OL group. At the end of the 8‐week intervention, the IHL level decreased to 11.1% ± 1.6% in the canola oil group but increased to 15.7% ± 2.7% in the olive oil group. This reduction in IHL was significantly greater in the canola oil group than in the olive oil group (*p* = 0.038). In Nigam et al. (2014) study, the distribution of hepatic steatosis grades among participants was assessed before and after the intervention. In the OL group, 22 patients were Grade I, 6 were Grade II, and 2 had Grade III hepatic steatosis before intervention. Postintervention, the number of patients with Grade I steatosis decreased to 7, whereas the number of Grade II and Grade III cases decreased to 3 and 0, respectively. A total of 20 patients were classified as having normal liver imaging results after the intervention. In the CA group, preintervention, 20 patients had Grade I steatosis, 11 had Grade II steatosis, and 2 had Grade III steatosis. Postintervention, the number of Grade I cases decreased to 9, Grade II cases to 1, and Grade III cases to 0. A total of 20 patients were classified as having normal liver imaging results after the intervention. In the RA group, before intervention, 17 patients had Grade I steatosis, 9 had Grade II steatosis, and 4 had Grade III steatosis. Postintervention, the number of Grade I cases increased to 19, while the number of Grade II cases slightly decreased to 8, and the number of Grade III cases decreased to 0. Only 3 participants were classified as having normal liver imaging results. The reduction in hepatic steatosis grade observed in both the OL and CA groups, particularly in the Grade II and normal liver categories, was statistically significant compared with that in the RA group (*p* < 0.01). In Sedgi's study, in the CA group, there were no patients with normal liver imaging or Grade I steatosis before the intervention. However, by the end of the 12th week, 8 patients were classified as normal, and 31 patients had Grade I steatosis. Additionally, the number of Grade II patients decreased from 48 to 16, whereas Grade III patients were eliminated. The rate of steatosis grade reduction was 41.81%, whereas 8.18% of patients showed no change. Similarly, in the ghee group, there were no patients with normal liver imaging or Grade I steatosis before the intervention. The group initially included 46 patients with Grade II steatosis and 9 patients with Grade III steatosis. After 12 weeks of intervention, the number of normal patients remained at 0, while 10 patients had Grade I steatosis, 39 had Grade II steatosis, and 6 had Grade III steatosis. The rate of steatosis reduction in this group was 13.63%, with 36.54% of patients showing no change and 1.81% experiencing deterioration. The changes in hepatic steatosis levels in both the CA and ghee groups were statistically significant (*p* < 0.001).

### 3.7 Effects of Canola Oil on Liver Enzymes

A meta‐analysis including four intervention arms from three randomized controlled trials was conducted for ALT and AST levels. Heterogeneity analysis showed *Q* = 2.803 (*p* = 0.423) and *I* ^2^ = 0% for ALT, and *Q* = 7.538 (*p* = 0.057) and *I* ^2^ = 60.2% for AST. Random‐effects models were applied for all pooled analyses.

**FIGURE 3:**
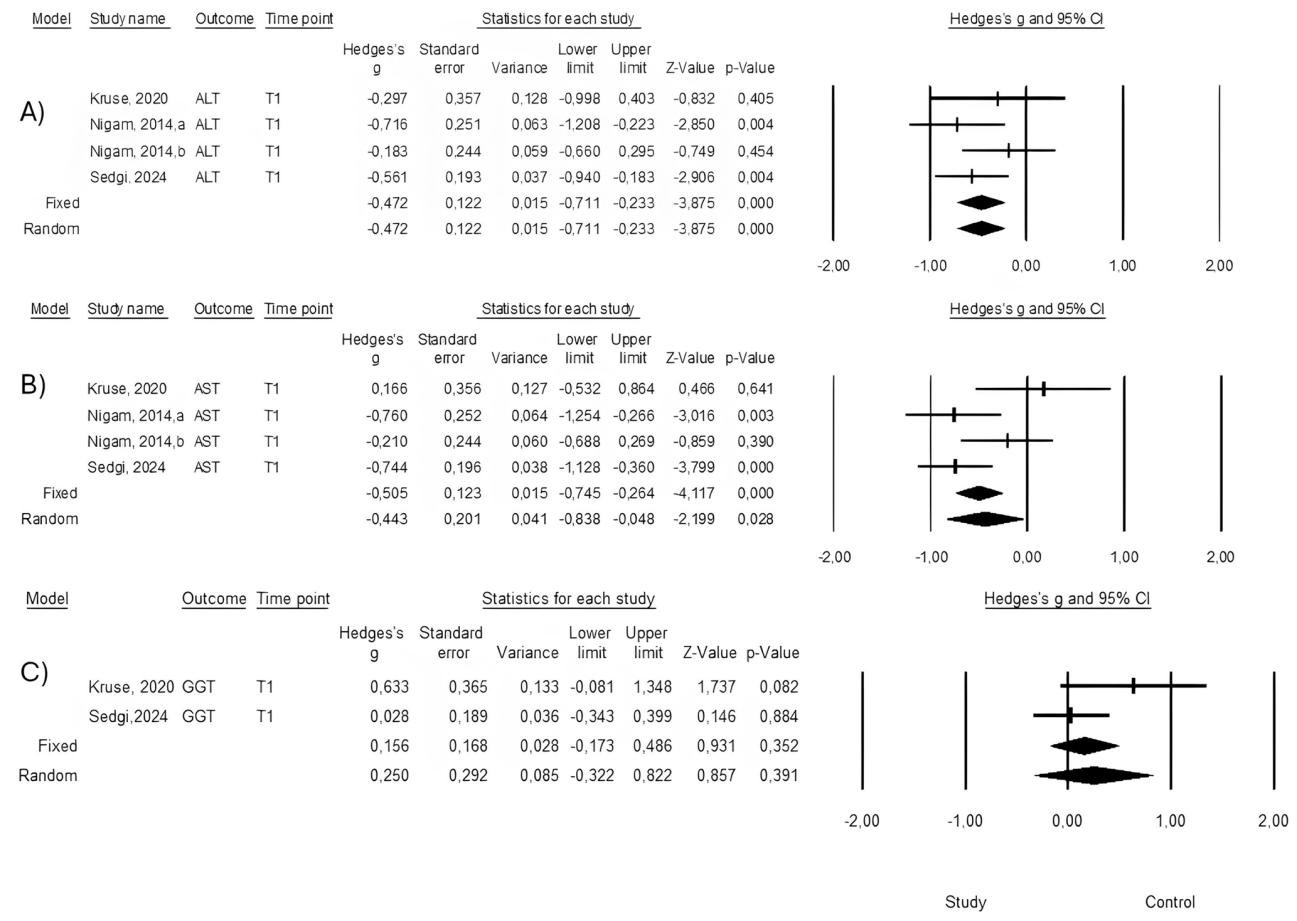
Forest plots showing the pooled effects of canola oil on (A) ALT, (B) AST, and (C) GGT levels in adults with NAFLD. Effect sizes were calculated using random‐effects models and are presented as standardized mean differences (SMDs) with 95% confidence intervals. Negative values indicate greater reductions in the canola oil group compared with comparator oils.

The pooled effect size for ALT was −0.472 (95% CI: −0.711 to −0.233; *p* < 0.001), indicating a statistically significant reduction. For AST, the pooled effect size was −0.505 (95% CI: −0.745 to −0.264; *p* < 0.001), also demonstrating a significant decrease (Figure 3).

A leave‐one‐out influence analysis was conducted to assess robustness. For ALT, removal of individual studies resulted in effect sizes ranging from −0.493 to −0.397, with all iterations maintaining statistical significance, indicating stable findings. For AST, effect estimates ranged from −0.584 to −0.308, with the direction of association remaining consistent across iterations, although statistical significance varied in some cases. These findings suggest that ALT results are robust, whereas AST estimates should be interpreted with moderate caution.

For triglycerides, the pooled estimates ranged from −0.714 to −0.310, with most iterations maintaining the negative direction; however, *p*‐values fluctuated considerably. For GGT and HDL, both based on only three studies, the analyses showed substantial sensitivity to individual study removal. Overall, these analyses demonstrate that while ALT findings are robust, biomarkers represented by fewer studies require cautious interpretation. The complete leave‐one‐out influence matrix is presented in Table 1. Due to the small number of included studies, publication bias could not be reliably assessed.

A meta‐analysis including two studies evaluated the effect of canola oil on GGT levels. Heterogeneity analysis yielded *Q* = 2.172 (*p* = 0.141) and *I* ^2^ = 53.9%. Using a random‐effects model, the pooled effect size was 0.156 (95% CI: −0.173 to 0.486; *p* = 0.352), indicating no statistically significant effect.

### 3.8 Effects of Canola Oil on Blood Lipids

**FIGURE 4:**
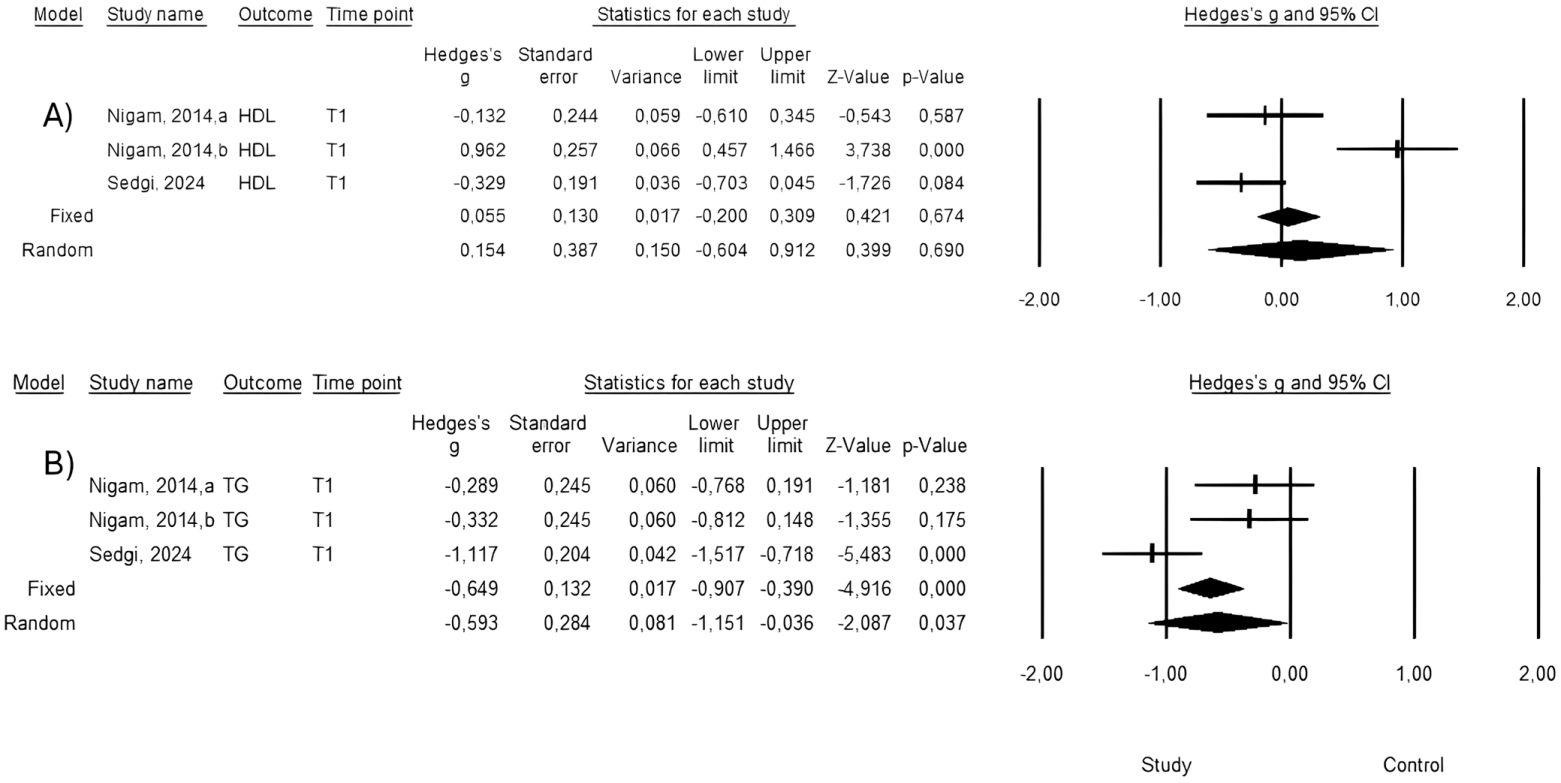
Forest plots showing the pooled effects of canola oil on (A) HDL cholesterol and (B) triglyceride (TG) levels in adults with NAFLD. Random‐effects models were applied. Effect sizes are presented as standardized mean differences (SMDs) with 95% confidence intervals. Negative values favor canola oil for triglycerides, whereas positive values favor canola oil for HDL.

Three studies were included in the meta‐analysis of HDL and triglyceride (TG) levels. Substantial heterogeneity was observed (HDL: *Q* = 17.068, *p* < 0.001; *I* ^2^ = 88.3%; TG: *Q* = 9.124, *p* = 0.01; *I* ^2^ = 78.1%). Therefore, random‐effects models were applied. The pooled effect size for HDL was 0.154 (95% CI: −0.604 to 0.912; *p* = 0.69), indicating no significant effect. For TG, the pooled effect size was −0.593 (95% CI: −1.151 to −0.036; *p* = 0.037), demonstrating a statistically significant reduction (Figure 4).

Given the limited number of included studies, formal statistical tests for publication bias were not considered reliable. Leave‐one‐out analyses indicated that TG results varied in magnitude across iterations, although the direction of effect remained predominantly negative.

## 4 Discussion

This systematic review and meta‐analysis evaluated three randomized controlled trials, including a total of 220 participants, with a focus on the effects of canola oil intervention on liver health. These findings suggest that canola oil consumption has beneficial effects on liver enzymes such as AST and ALT. Although statistically significant reductions in ALT and AST levels were observed, the clinical relevance of these changes should be interpreted with caution. Aminotransferases are biochemical markers of hepatocellular injury and do not necessarily reflect histological improvement or fibrosis regression. The effect sizes identified in this meta‐analysis suggest modest biochemical improvement rather than definitive structural recovery of the liver. Therefore, while these findings may indicate a potential supportive role of dietary canola oil in reducing hepatic metabolic stress, they should not be considered evidence of long‐term disease modification in NAFLD. The trends in ALT, AST, and triglyceride improvement observed in this meta‐analysis align with high‐impact reviews suggesting that canola oil modulates hepatic lipid turnover and inflammatory signaling (Farhangi et al. 2022; Musazadeh et al. 2021, 2022). Such evidence strengthens the biological plausibility of our results and highlights the clinical relevance of dietary fat composition in NAFLD management. Additionally, elevated GGT levels have been linked to cardiovascular diseases, with evidence suggesting associations with early‐onset coronary artery disease (Xuan et al. 2023) and acute coronary syndrome prediction in type 2 diabetes patients (Al‐Suhaimi and Al‐Rubaish 2024). However, no significant effects on GGT levels were detected. Elevated GGT levels are associated with hepatic insulin resistance and increased intrahepatic lipid accumulation (Thamer et al. 2005).

The effects of canola oil on serum lipids could only be assessed on the basis of studies by Nigam and Sedgi, which involved a total of 203 participants. Canola oil consumption was associated with reductions in serum TG levels. These findings suggest that canola oil may contribute to improvements in biochemical markers associated with NAFLD, a key pathological process in NAFLD development. Individual trials reported reductions in liver steatosis; however, differences in assessment methods precluded quantitative pooling. Amiri et al. reported that canola oil has beneficial effects on the TC, LDL, Apo B, TC/HDL, LDL/HDL, and Apo B/Apo A‐1 ratios (Amiri et al. 2020). These potential risks reinforce the need for balanced oil consumption and careful consideration of heat stability, particularly when applying dietary recommendations for NAFLD. Although triglyceride levels were significantly reduced in the pooled analysis, the degree of statistical heterogeneity was substantial, and sensitivity analyses indicated variability across individual studies. These factors indicate that the observed triglyceride reduction should be interpreted as suggestive rather than definitive. Differences in intervention dose, duration, comparator oils, and baseline metabolic characteristics may have contributed to the variability in effect estimates.

Despite the positive effects on TG levels, no statistically significant effect on HDL levels was observed. However, a notable increase in HDL was reported in a study comparing canola oil with safflower/soybean oil (Nigam 2014b). This discrepancy may be attributed to individual variations, differences in intervention dosages, or treatment durations. Moreover, compared with olive oil, canola oil is not expected to have a significantly greater effect on HDL levels. Leave‐one‐out sensitivity analysis did not materially change the direction of the pooled estimate, and HDL remained non‐significant across all iterations. Nevertheless, Pourrajab et al. (2023) reported that compared with olive oil, canola oil has beneficial effects on LDL, TC, and the LDL/HDL ratio, suggesting its potential cardioprotective properties. Although LDL levels could not be assessed in this study, several meta‐analyses reported that canola oil consumption has a positive effect on total cholesterol and LDL (Ghobadi et al. 2019; Jafari Azad et al. 2020; Schoeneck and Iggman 2021; Voon et al. 2024). Interestingly, the metabolic effects of canola oil appeared comparable to those of olive oil, suggesting that oil quality rather than specific oil type may be more relevant in the context of NAFLD.

Given the heterogeneous metabolic responses to dietary fats, these findings underscore the importance of precision nutrition approaches in tailoring oil recommendations for individuals with NAFLD.

It is important to interpret these findings in light of the specific comparator oils used in the included trials. Olive oil, safflower/soybean oil, and ghee differ substantially in their fatty acid composition and metabolic effects. Therefore, the observed changes cannot be attributed solely to canola oil itself but rather to its relative composition compared with the alternative fat source. For instance, comparisons with olive oil primarily reflect differences within unsaturated fat profiles, whereas comparisons with ghee involve replacing saturated fat‐rich sources. These distinctions may partly explain variations in lipid and hepatic outcomes across studies, highlighting the importance of contextualizing dietary fat substitutions rather than referring broadly to control oils. NAFLD is closely linked to increased cardiovascular risk and has been associated with higher carotid intima–media thickness (CIMT), even after adjustment for traditional risk factors (Tarantino et al. 2014). Previous meta‐analyses have shown that canola oil may improve LDL cholesterol and other cardiometabolic markers due to its favorable fatty acid composition. Although the triglyceride reductions observed in this analysis may suggest a potential cardiometabolic benefit, none of the included trials assessed direct vascular outcomes such as CIMT or endothelial function. Therefore, conclusions regarding atherosclerotic progression or cardiovascular protection remain speculative and require confirmation in long‐term studies incorporating vascular endpoints.

In conclusion, evidence from a limited number of randomized controlled trials suggests that dietary canola oil may lead to modest improvements in hepatic enzymes and triglyceride levels in adults with NAFLD. Therefore, while canola oil may represent a reasonable unsaturated fat option within dietary strategies for NAFLD, larger and longer‐term trials are required to determine its clinical impact on disease progression and cardiometabolic risk.

## 5 Strengths

This study was conducted following the PRISMA and Cochrane guidelines and was verified via the PRISMA checklist. The risk of bias in the included studies was assessed via the Cochrane risk of bias tool. Statistical analyses were performed with Comprehensive Meta‐Analysis V3.3.070. Heterogeneity was quantified using standard statistical metrics (*Q* and *I* ^2^). In addition, the certainty of evidence was formally graded using the GRADE approach, and a comprehensive leave‐one‐out influence analysis was performed to confirm the robustness and stability of the pooled estimates.

## 6 Limitations

This study has several limitations that should be acknowledged. The number of included randomized controlled trials was limited, resulting in relatively small pooled sample sizes and reduced statistical power. Substantial clinical heterogeneity existed across studies, including differences in intervention dose (20–50 g/day), duration (8 weeks to 6 months), comparator oils (olive oil, safflower/soybean oil, and ghee), and study design (replacement vs. supplementation approaches). And liver fat was assessed using different methodologies (^1^H‐MRS vs. ultrasonography), which vary in sensitivity and precision. This methodological variability may have influenced confidence in steatosis‐related outcomes. Furthermore, some trials were open‐label in design, and certain study populations were restricted (e.g., male‐only cohorts), limiting generalizability. Furthermore, intervention durations were relatively short, and no study evaluated long‐term clinical outcomes such as fibrosis progression, cirrhosis development, or cardiovascular events.

## Author Contributions

**Kardelen Büşra Ege Gündüz:** conceptualization, investigation, methodology, software, data curation, formal analysis, funding acquisition, writing – original draft, writing – review and editing, visualization, project administration, resources. **Sümeyye Bora:** participated in the study screening process, provided feedback and contributed to manuscript writing. **Berrak Baştürk:** extracted and analysed the data, contributed to manuscript writing, provided feedback, and approved the final version. **Ceyda Okudu:** designed the research, contributed to final editing and revisions, provided feedback, and approved the final version.

## Funding

The authors have nothing to report.

## Ethics Statement

The authors have nothing to report.

## Consent

All authors have approved the version of the manuscript submitted and agreed to publish this manuscript in Food Science & Nutrition. All the authors declare that this study is not published or submitted elsewhere for peer review.

## Conflicts of Interest

The authors declare no conflicts of interest.

## Supporting information


**Appendix S1:** PRISMA checklist.

**Appendix S2:** Search strategy.

**Table S1:** Elegibility criteria for the systematic review according to PICOS framework.

## Data Availability

The data that support the findings of this study are available upon request from the corresponding author.
