# Supplementary material for: Effects of Canola Oil on Hepatic and Cardiometabolic Markers in Non‐Alcoholic Fatty Liver Disease: A Systematic Review and Meta‐Analysis
Source: Food Sci Nutr. 2026 Apr 19;14(4):e71752. doi: 10.1002/fsn3.71752 (PMC13092728; doi:10.1002/fsn3.71752)
Supplement: Supplementary file 2 — Appendix S2: Search strategy. [file FSN3-14-e71752-s003.docx]

**Full search strategies for each of the databases (1-16 Dec 2024)**

**Pubmed: 356**

((((((canola oil) OR (rapeseed oil)) OR (low erucic acid rapeseed)) OR (LEAR)) OR (colza oil)) OR (Brassica napus)) AND (((((((canola oil) OR (rapeseed)) OR (low erucic acid rapeseed)) OR (LEAR)) OR (colza oil)) OR (Brassica napus)) AND ((((((NAFLD) OR (Non alcoholic fatty liver)) OR (Steatohepatitis)) OR (Steatohepatitides)) OR (Fatty liver)) OR (NASH)))

**Cochrane: 21**

**#1** (canola oil OR rapeseed oil OR low erucic acid rapeseed OR LEAR OR colza OR Brassica napus):ti,ab,kw

**#2** NAFLD OR Nonalcoholic Fatty Liver OR Steatohepatitis OR Steatohepatitides OR Fatty liver OR NASH

**#3** #1 AND #2

**Web of Science = 713**

1. ALL= (canola oil OR rapeseed oil OR low erucic acid rapeseed OR lear OR colza oil OR brassica napus**)**

2. ALL= (NAFLD OR Nonalcoholic Fatty Liver OR Steatohepatitis OR Steatohepatitides OR Fatty liver OR NASH)

3. #1 AND #2

**Scopus =80**

TITLE-ABS-KEY("canola oil" OR "rapeseed oil" OR "low erucic acid rapeseed" OR lear OR "colza oil" OR "brassica napus" ) AND TITLE-ABS-KEY(NAFLD OR "Nonalcoholic Fatty Liver" OR Steatohepatitis OR Steatohepatitides OR "Fatty liver" OR NASH)
