# Supplementary material for: Effects of Canola Oil on Hepatic and Cardiometabolic Markers in Non‐Alcoholic Fatty Liver Disease: A Systematic Review and Meta‐Analysis
Source: Food Sci Nutr. 2026 Apr 19;14(4):e71752. doi: 10.1002/fsn3.71752 (PMC13092728; doi:10.1002/fsn3.71752)
Supplement: Supplementary file 3 — Table S1: Elegibility criteria for the systematic review according to PICOS framework. [file FSN3-14-e71752-s002.docx]

**PICOS Framework**

Supplementary table 3: Elegibility criteria for the systematic review according to PICOS framework.

| Population | People with NAFLD |
| --- | --- |
| Intervention | Canola oil consumption |
| Control | Other oils or fats (olive oil, safflower/soybean oil, ghee) |
| Outcomes | Liver AST-ALT levels  Blood lipid leves (Total cholesterol, Triglyceride, HDL, LDL) |
| Study Type | Randomized controlled trials, cohort studies, cross-sectional studies, observatory studies |
